# Hepatoblastoma in a child with dextrocardia and possible histopathological alteration reminiscent of hepatocellular carcinoma after neoadjuvant chemotherapy

**DOI:** 10.1002/ccr3.1515

**Published:** 2018-04-17

**Authors:** Afsana Papry, Mohammed Kamal, Muhammad Syeef Khalid

**Affiliations:** ^1^ Department of Pathology Bangabandhu Sheikh Mujib Medical University Shahbag Dhaka 1000 Bangladesh; ^2^ Department of Surgical Oncology National Institute of Cancer Research and Hospital Mohakhali Dhaka 1212 Bangladesh

**Keywords:** Hepatoblastoma, hepatocellular carcinoma, neoadjuvant chemotherapy

## Abstract

It is quite unambiguous and interesting that postchemotherapy histology of hepatoblastoma may mimicry that of hepatocellular carcinoma which should be differentiated by proper immunohistochemistry and cytology, if possible, for further management and predict prognosis.

## Background

Hepatoblastoma is the most common malignant liver tumor in children globally. Its presentation is quite common at early age. Nowadays, after inventing available effective chemotherapeutic agents, neoadjuvant chemotherapy is usually given in primarily unresectable tumor. But in a very few cases, after chemotherapy, the tumor may exhibit some property of hepatocellular carcinoma, that is, necrosis and fibrohistiocytic responses.

## Case Report

**Figure 1:**
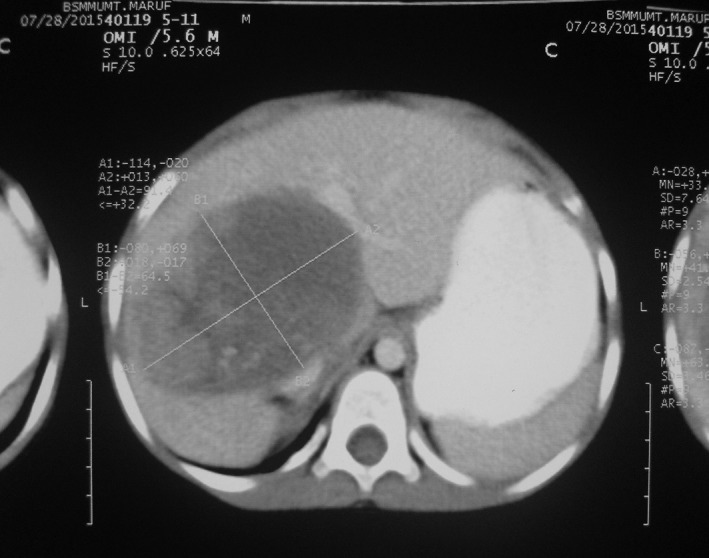
CT scan abdomen shows rounded hypodense area in right lobe of liver.

**Figure 2:**
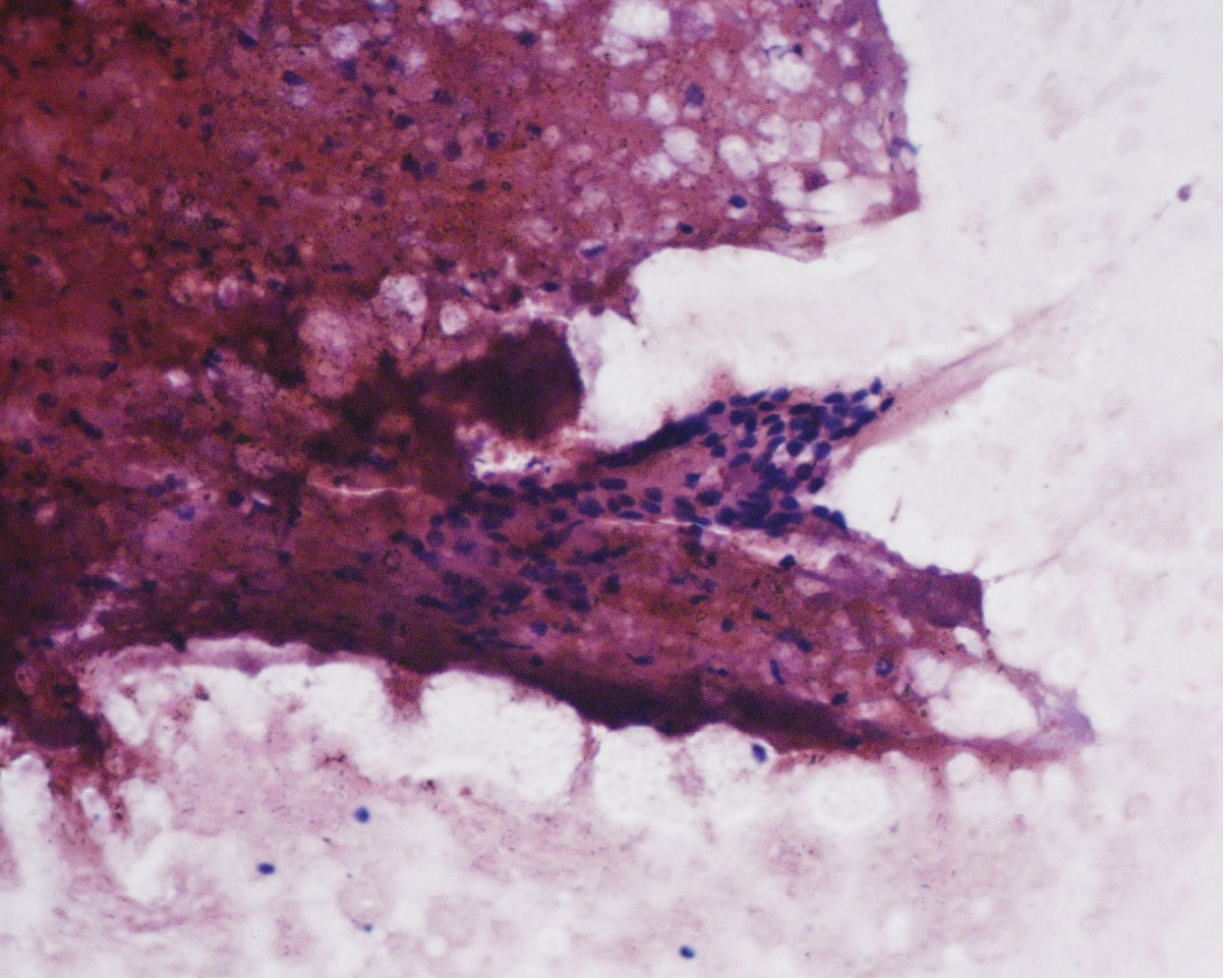
FNA shows small undifferentiated cells in a background of necrotic material.

**Figure 3:**
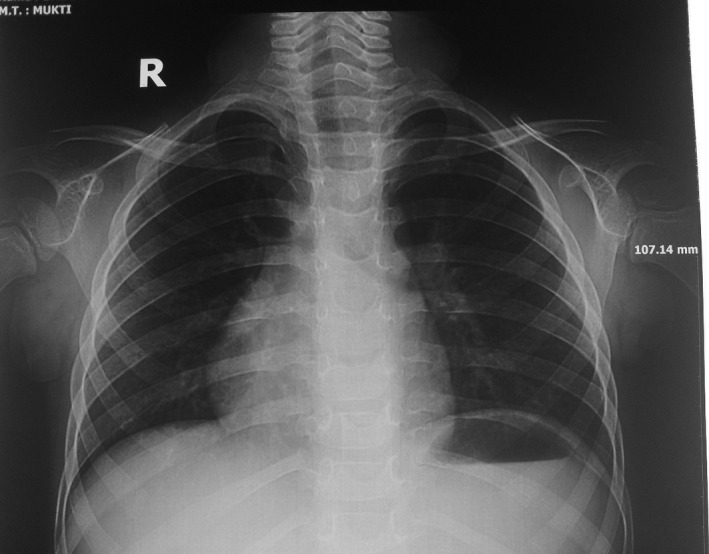
X‐ray chest PA view shows dextrocardia.

A 5‐year‐old boy, hailing from Dhaka, Bangladesh, presented to Bangabandhu Sheikh Mujib Medical University hospital with a painless upper abdominal lump. According to his mother's statement, the boy was well up to 4 years of age. Then, a lump in right upper abdomen was observed incidentally which was rapidly increasing in size but presented no symptoms at that time. The patient had no history of jaundice, cough, fever, or bone pain. His bowel–bladder habit was normal. After routine laboratory investigations, the mass was evaluated as a hepatic tumor. Serum alpha‐fetoprotein (AFP) level was >300,000.0 ng/mL (Reference Value: 1–10 ng/mL). Ultrasonography and computed tomography scan both reported hepatomegaly due to a large tumor suggestive of hepatoblastoma measuring about 9.1 cm × 6.4 cm which was present in the right lobe of liver involving segment VI, VII, and VIII with tiny calcifications (Fig. 1). Ultrasound‐guided FNA from SOL liver was carried out. The smears revealed few ovals to spindly cells with high nuclear cytoplasmic ratio. There were also few atypical small undifferentiated cells. The cytological diagnosis was suggestive of hepatoblastoma (Fig. 2). Both screening and confirmatory tests for hepatitis‐B were negative. Additionally, chest x‐ray reveals dextrocardia (Fig. 3). Other than this, no other congenital anomaly was found.

For this tumor, neoadjuvant chemotherapy was given by Department of Pediatric Oncology at BSMMU hospital, and 4th cycle was completed on June 24, 2015. The chemotherapeutic agents were Vincristine, Cisplatin, and 5‐FU. Right lobectomy of liver was carried out on August 18, 2015, by Department of Hepatic Surgery at BSMMU, and specimen was sent to Department of Pathology.

**Figure 4:**
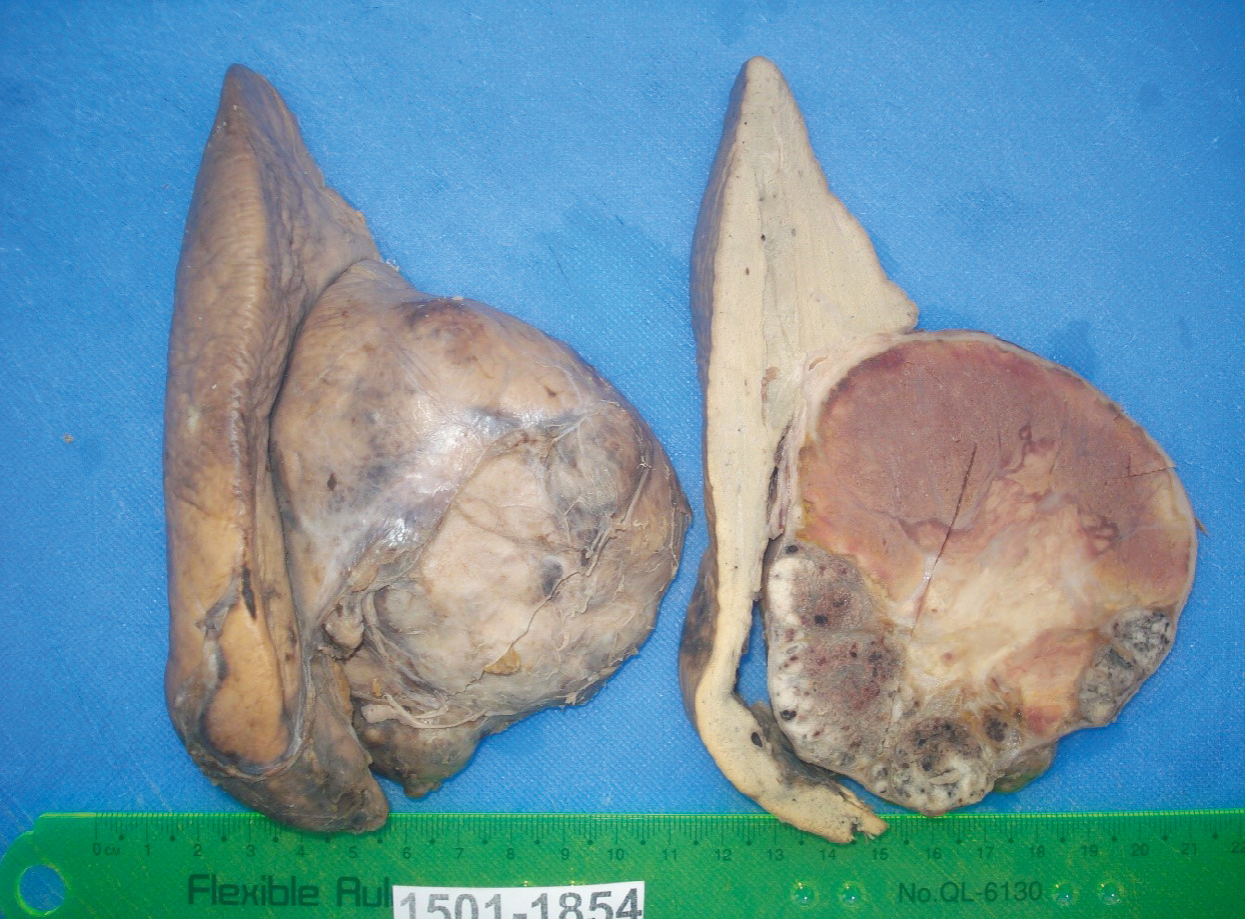
Liver mass (8.5 × 7 cm) in right lobe.

On gross examination, the specimen was recognized as right lobe of the liver. Sectioning revealed a well circumscribed and capsulated mass measuring 8.5 cm in maximum diameter. The cut surface of the mass was partly hemorrhagic and partly gray white (Fig. 4).

**Figure 5:**
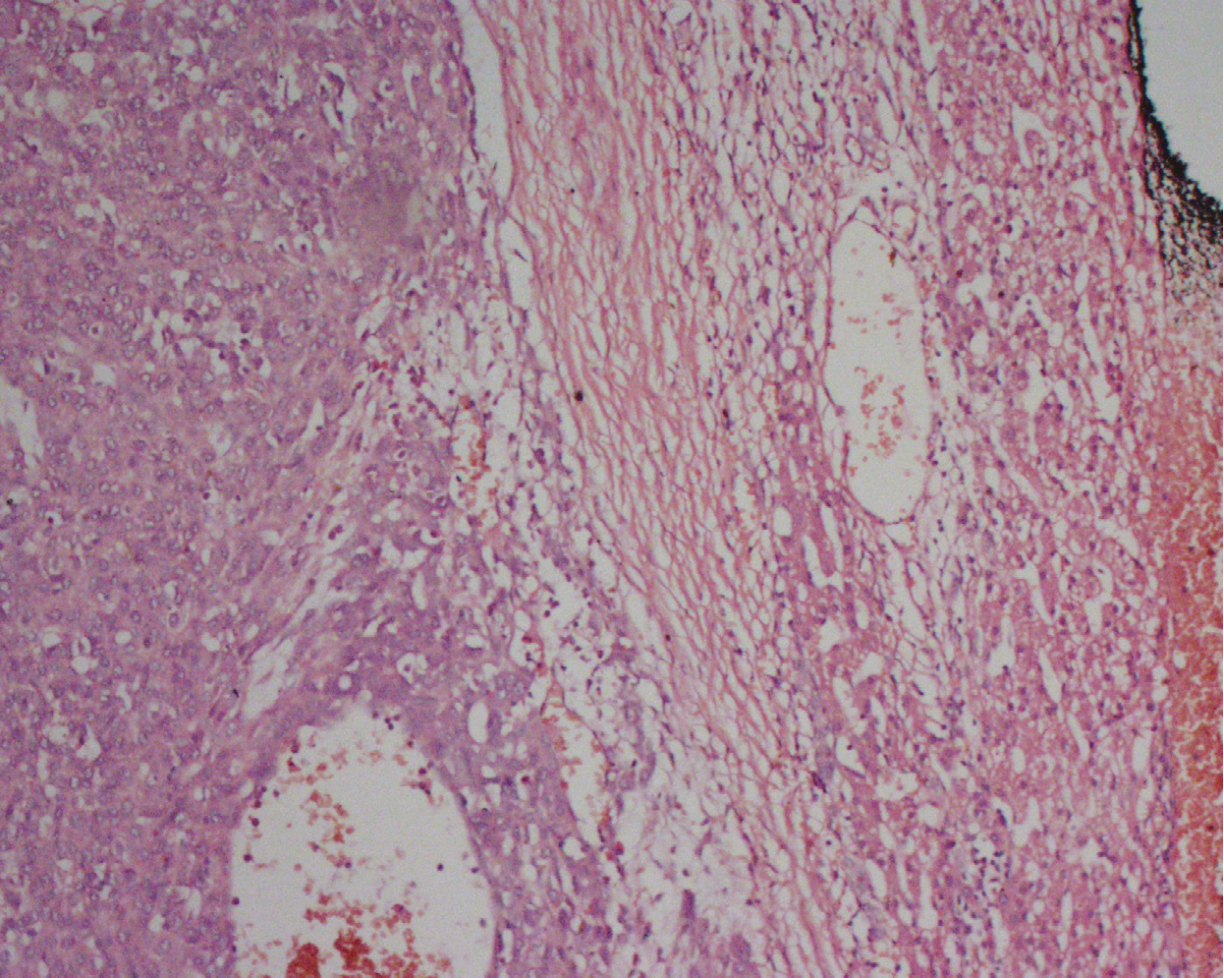
Malignant tumor reveals hepatocellular carcinoma.

**Figure 6:**
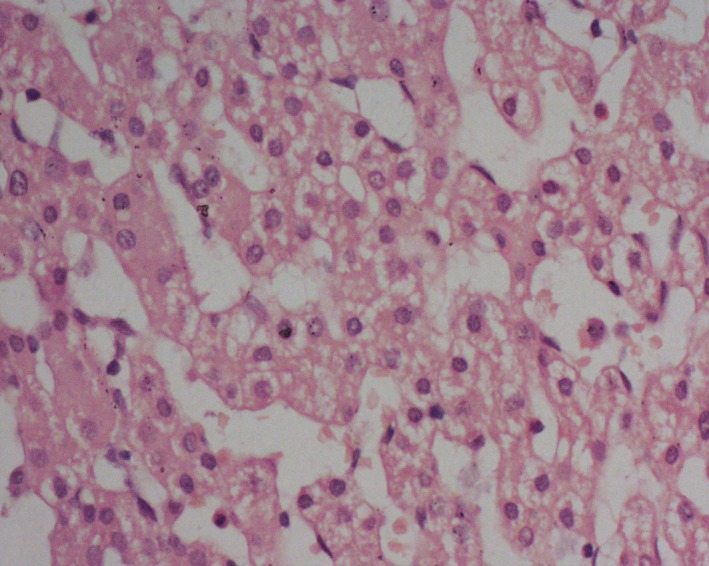
Malignant hepatocytes arranged in trabecular pattern.

Routine hematoxylin‐ and eosin‐stained sections of the hepatic mass revealed mostly necrosed and hyaline areas with a few foci of viable area. The tumor microscopically had features of hepatocellular carcinoma (Figs. 5 and 6). The neoplastic cells had prominent nucleoli with occasional intranuclear pseudoinclusion. Some of the tumor cells have small amount of bile pigments in their cytoplasm. No fetal hepatocytes were seen. All resection margins were free of tumor. The postoperative period was uneventful, and the patient was released in due time. He was followed up regularly. The last follow‐up was in December 2015 at which the patient was doing well. His AFP level was within normal range.

## Discussion

Hepatoblastoma (HB) is the most common primary liver tumor in children, but isolated incidences in older children and adults have been reported 1, 2, 3. Hepatoblastoma accounts for 79% of all liver tumors in children and almost two‐thirds of primary malignant liver tumors in the pediatric age group 4. Four percent of hepatoblastomas are present at birth, 68% in the first 2 years of life, and 90% by 5 years of age. Only 3% are seen in patients over 15 years of age 1. Interestingly, incidence of hepatocellular carcinoma is quite low in children, accounting for approximately 0.5–2% of all neoplasms in these age groups. Annual incidence in children is approximately 0.5 cases per million 4. US Surveillance, Epidemiology and End Results report showed of 271 primary hepatic malignancies reported between 1973 and 1997, the group under 5 years of age had hepatoblastoma in 90% of cases 5. The tumor has been seen in association with a variety of anomalies including congenital abnormalities (particularly hemihypertrophy of liver), Wilms tumor of kidney, glycogen storage disease, dextrocardia, Aicardi syndrome, Beckwith–Wiedemann syndrome, low‐birth‐weight infants, and familial colonic polyposis 5. There is a male predominance of 1.5:1 to 2:1 1.

Most of the patients present with an enlarging abdominal mass. The right lobe is involved three times more commonly than the left, with bilobar involvement seen in 20–30%, and multicentric involvement in 15% cases 6, 7. Bilirubin and liver enzymes are usually normal. Anemia and platelet abnormalities have been reported. Serum AFP level is almost always elevated which is a sensitive marker for the progression of the disease 6. Besides, imaging plays an immense role in the diagnosis. Traditional imaging modalities (conventional radiography, excretory urography, and hepatic arteriography) have largely been replaced by ultrasonography, spiral CT, and MRI. Calcification may be seen by CT in more than 50% of cases 3.

Macroscopically, hepatoblastomas vary in size from commonly 5 to 22 cm in diameter and from 150 to 1400 g in weight 1. It is solid, well circumscribed, and more often solitary than multiple. The tumors are well circumscribed and may be partially encapsulated, nodular, with a bulging cut surface. A cystic component may be present, and areas of necrosis and hemorrhage are frequent. The color may range from tan‐brown to green to white, depending on the presence of variable subtypes 1, 2, 6.

Hepatoblastoma is subclassified histologically into six histologic patterns 1. These are pure fetal epithelial differentiation (31%), combined fetal and embryonal epithelial (20%), macrotrabecular (3%), small cell undifferentiated (3%), mixed epithelial and mesenchymal (43%) and Mixed with teratoid features.

The main histopathological components of HB are epithelial and mesenchymal; both are present in varying proportions and at various stages of differentiation. The epithelial element recapitulates the stages of hepatocyte development, whereas the blastemal or undifferentiated cells have been postulated to represent neoplastic hepatocyte progenitor cells 5.

In our case, the diagnosis of hepatoblastoma was made on clinical, radiological, and FNA findings. Unfortunately, we could not subtype the tumor because of scanty cells in FNA, and no residual hepatoblastoma like structure was present in resected specimen after neoadjuvant chemotherapy.

Wang et al. 8 showed in a 15‐year retrospective study of HBs in 22 children who received neoadjuvant chemotherapy according to the Children's Oncology Group protocols that treated HBs had necrosis and fibrohistiocytic response. Besides, two‐thirds had areas of cytoarchitectural differentiation (“maturation”) resembling non‐neoplastic liver, and a quarter had alterations mimicking hepatocellular carcinoma. Rosai and Desmet 2 claim that neoadjuvant chemotherapy can induce cytoarchitectural differentiation mimicking non‐neoplastic liver (but can be distinguished from the latter by nuclear expression of ß‐catenin) and alterations reminiscent of hepatocellular carcinoma.

In this case, we found no fetal hepatocytes after chemotherapy because of gross fibrohistiocytic response. Morphological features resembled with that of hepatocellular carcinoma. We also found neoplastic cells with large nucleoli having occasional intranuclear pseudoinclusion formed by cytoplasmic invaginations which is a classical feature of HCC. Some of the tumor cells exhibited small amount of bile pigments in their cytoplasms which is another diagnostic feature of HCC.

Heifetz et al. 9 reported that vascular invasion, amount of mesenchyme, persistence of embryonal epithelium, extent of tumor necrosis, and mitotic activity of the epithelial component have predictive value in this type of specimen.

Saxena et al. 6 reported in a 11‐year‐long study on 17 children with HB to have osteoid in more than 82% cases. Interestingly, we found no osteoid in this case.

Different types of hepatoblastoma show affection for different immunoreactant or antibody. Immunohistochemically, reactivity has been found for keratin, epithelial membrane antigen (EMA), vimentin, polyclonal CEA, HepPar‐1, AFP, a_1_‐antitrypsin, CD99, CD56 (N‐CAM), human chorionic gonadotropin, transferrin receptor, and delta‐like protein. On the contrary, liver cell carcinoma has been found to be immunoreactive for AFP, pan‐keratin, EMA, a_1_‐antitrypsin, HepPar‐1, etc. Although, these antibodies vary in sensitivity and specificity with a high cost‐benefit ratio, can be used effectively to differentiate hepatoblastoma from hepatocellular carcinoma 2. Cytology also can bolster the diagnosis, but that is out of scope of this review.

## Conclusion

Due to rarity of the disease, many aspects of diagnosis and treatment of hepatoblastoma are still under research and evolution. However, it is quite unambiguous and interesting that postchemotherapy histology of hepatoblastoma may mimicry that of hepatocellular carcinoma which should be differentiated by proper immunohistochemistry and cytology, if possible, for further management.

## Conflict of Interest

None declared.

## Authorship

AP: Involved in making the diagnosis by studying the histopathological and cytopathological slides. MK: Involved in making the diagnosis by studying the slides as a direct supervisor and contributed to scholarly discussion and guidance. MSK: Involved in literature review, workup, and compilation of the whole of the topic.
